# Health care workers’ self-perceived meaning of residential care work

**DOI:** 10.1186/s12913-024-11218-2

**Published:** 2024-06-26

**Authors:** Sui Yu Yau, Yin King Linda Lee, Siu Yin Becky Li, Sin Ping Susan Law, Sze Ki Veronica Lai, Shixin Huang

**Affiliations:** 1 Hong Kong Metropolitan University, Jockey Club Institute of Healthcare, 1 Sheung Shing Street, Homantin, Hong Kong; 2 The Hong Kong Polytechnic University, Hung Hom, Kowloon, Hong Kong

**Keywords:** Health care work, Long-term care workforce, Meaning of work

## Abstract

**Background:**

Attracting and supporting a sustainable long-term care (LTC) workforce has been a persistent social policy challenge across the globe. To better attract and retain a sustainable LTC workforce, it is necessary to adopt a unified concept of worker well-being. Meaning of work is an important psychological resource that buffers the negative impacts of adverse working conditions on workers’ motivation, satisfaction, and turnover intention. The aim of this study was to explore the positive meaning of care work with older people and its implications for health care workers’ job satisfaction and motivation to work in the LTC sector.

**Methods:**

This study adopted a qualitative descriptive design that pays particular attention to health care workers; such as nurses, personal care workers; as active agents of the meaning making and reframing of care work in LTC communities in a East Asia city. In-depth semi-structured interviews were conducted with thirty health care workers in LTC communities in Hong Kong. Thematic analysis was employed for data analysis.

**Results:**

The research findings indicate that while health care workers perform demanding care work and experience external constraints, they actively construct positive meanings of care work with older people as a helping career that enables them to facilitate the comfortable aging of older people, build affectional relationships, achieve professional identity, and gain job security.

**Conclusions:**

This qualitative study explores how health care workers negotiate the positive meaning of older people care work and the implications of meaningful work for workers’ job satisfaction and motivation to work in the LTC sector. The importance of a culturally sensitive perspective in researching and developing social policy intervention are suggested.

**Supplementary Information:**

The online version contains supplementary material available at 10.1186/s12913-024-11218-2.

## Introduction

Recruiting and retaining health care workers (HCWs) in the long-term care (LTC) sector is a persistent worldwide social policy challenge [1]. Across the globe, population aging will create significantly higher demands for LTC services for older people. These demands include residential care services, especially among older people with complex care needs due to age-related disabilities and chronic diseases [2]. Comprised mainly of nurses and personal care workers, HCWs in LTC communities perform a variety of tasks that are essential to maintain the functional ability of older people, including helping with activities of daily living (ADL) (such as bathing, toileting and eating), instrumental activities of daily living (IADL) (such as taking medication), monitoring and coordinating care, and communicating with older people and their families [3]. Despite the growing demand and significance of LTC services, health care work in LTC communities is often devalued as “dirty work” and characterized by low wages, precarious working conditions, limited career development opportunities, understaffing, and work overload [4].

In the context of LTC communities, while the research to date has extensively evaluated the demanding working conditions that lead to negative well-being outcomes for HCWs [1], relatively little is known about the positive meaning that HCWs experience in, and attribute to, their care work in LTC communities [5]. Further exploration of how HCWs engage in meaningful work is helpful to the development of strategies that improve worker well-being and other work outcomes in LTC communities, especially job satisfaction and worker retention. In addition, cultural and social contexts exert a heavy influence on the meaning of care [6]. Most of the current literature on older people care work has been produced and addressed in Anglo-American contexts; there are limited evaluations of the meanings and experiences of older people care work from the perspectives of HCWs in East Asia, a region that is characterized by a large, rapidly aging population and unique socio-cultural meanings of older people care. A culturally sensitive understanding of what contributes to meaningful work in the LTC setting is thus needed to attract and support the LTC workforce beyond the Western contexts. Thus, this qualitative study aims to examine how HCWs in LTC communities construct positive meanings of older people care and also the implications of meaningful work for their job satisfaction and intention to stay in the LTC sector in Hong Kong, in the People’s Republic of China. This study is produced as part of a larger research project examining the social construction of stigma attached to older people care work in Hong Kong’s LTC communities [7] and pays particular attention to HCWs’ meaning construction in relation to the policy, organizational, and socio-cultural contexts to inform LTC workforce development policy.

## Background

### Constructing meaning of work in LTC communities

Meaning of work (MOW) is an important psychological resource that buffers the negative impacts of adverse working conditions on workers’ motivation, satisfaction, and turnover intention [8, 9]. Across different occupational contexts, organizational scholars have consistently found that MOW is a significant aspect of workers’ subjective well-being and is associated with positive worker and organizational outcomes, including higher work engagement, organizational commitment, worker retention, and productivity [10]. MOW refers to “employees’ understandings of *what* they do at work as well as the *significance*of what they do” [11]. It captures how employees make sense of their experiences at work, as well as the role of work in the context of life [12]. MOW consists of three primary facets: positive meaning in work, meaning making through work, and greater good motivation [13]. Meaning in work concerns individuals’ subjective interpretations of experiences and interactions at work in terms of the values, attitudes, and beliefs that they see as intrinsic to the nature of their work and working relationships [10]. Meaning making through work involves the idea that work could serve as a critical avenue for meaning making in life, such as facilitating personal growth, deepening self-understanding, and attaining personal and professional identity [14]. Lastly, greater good motivation implies the perception that one’s work has positive impacts on the greater good, ranging from generating positive contributions to others to responding to the meaning of work [15].

Although MOW is experienced by individual employees as feelings and cognitions, a sociological perspective of MOW suggests that the meaning individuals ascribe to their work is constructed within an array of socially influenced worldviews regarding the value of their work activities [16]. Individuals’ meaning making of their jobs, roles, and selves at work is a dynamic process that is influenced by the social and interpersonal valuation and devaluation of their work [11]. Work in the LTC sector is often socially constructed as “dirty work” that is physically, socially, and morally tainted [17, 18]. The social discourses on “dirty work” are further reinforced by the emotionally and physically demanding nature of care work, as well as the poor job quality in the LTC sector [19]. Work in LTC communities is typically characterized by poor compensation, heavy workloads, precarious part-time employment, limited career development prospects, limited training and supervision, and low occupational status compared to other healthcare fields [20].

Given these external constraints, it is not surprising that HCWs in LTC communities feel disempowered to make positive sense of their care work [21], which in turn negatively influences their job satisfaction and intention to work in the LTC sector [17]. Despite the social devaluation and demanding nature of older people care work, HCWs in LTC communities could actively engage in negotiating the meaning of their work and construct positive career identities to overcome the taint of dirty work, a research theme that to date remains underdeveloped [22]. These positive meanings might include forming caring relationships with older people [5].

### Residential care services and LTC workforce in Hong Kong

Health care workers in LTC communities negotiate the meaning of care work within particular social policy, organizational, and socio-cultural contexts [7]. Given the drastically increasing demand for residential care among older people, the chronic workforce crisis in the LTC sector, and the transforming socio-cultural meaning of care for older people [4], it has never been timelier to explore the meaning of work in Hong Kong’s LTC communities.

Hong Kong is an economically advanced metropolis located in the Southern part of China. With increasing life expectancy, Hong Kong’s aging population is projected to increase from 1.12 million (or 15% of total population) in 2015 to 1.51 million (or 30.6% of total population) in 2043, significantly higher than the OECD (Organisation for Economic Co-operation and Development) average percentage (25% in 2043) [23]. As a result, the demand for LTC services, including residential care services, will also increase drastically. The limited residential spaces, the transformation of family structure, and the imbalanced public investment in community and residential care have turned the number of older people who require residential care in Hong Kong into one of the highest among developed economies [24].

Hong Kong adopts a hybrid model in the financing and provision of its residential care services. In 2022, there were about 76,200 older people require residential care in Hong Kong, among which 46% (or a total number of 35,040) were subsidized by the government and 54% (or a total number of 41,160) were non-subsidized [25]. While residential care services in general are provided by non-governmental organizations (NGOs) (31%) and the private sector (69%), the majority of subsidized residential services are provided by NGOs, although the government also purchases subsidized places and services from private facilities [26]. Like many developed economies, Hong Kong has experienced an acute shortage of HCWs in LTC communities [26]. Even though the Hong Kong government has initiated many measures over the past few years to tackle the issues of the care workforce crisis, such as increasing salaries, launching different schemes to train young people and encouraging migrant workers to join the LTC communities, 20% of HCW positions in LTC communities remain vacant [27].

HCWs’ well-being is indeed connected to workforce attraction and retention. Despite the Hong Kong government initiating various ongoing measures to increase the number of workforce in LTC sector, there will be a shortfall of 4,500 HCWs in the next three year [28]. To better attract and retain a sustainable LTC workforce, it is necessary to adopt a unified concept of worker well-being that not only addresses the structural factors, such as economic and physical working conditions, but also the subjective factors that attract and motivate workers to join and remain in the LTC sector, including promoting meaningful, valued work [29, 30]. Caring for older people entails unique socio-cultural meanings in Hong Kong and East Asian societies. Although sociodemographic changes have transformed the patterns of social care for older people, most noticeably exemplified by the rising demand for residential care, such cultural norms still exert significant influences on the meaning of care work [31]. The aim of this study was to explore the positive meaning of care work with older people and its implications for health care workers’ job satisfaction and motivation to work in the LTC sector.

## Methods

This study adopted a qualitative descriptive design that focuses on HCWs as active agents of the meaning making and reframing of care work in LTC communities. The use of qualitative descriptive design is common in health care research because of its simplicity and flexibility in diverse healthcare environment. Qualitative research is appropriate to explore experiences and perceptions on subjective nature of a phenomenon. It is especially suitable for nursing and healthcare studies that interested in individual’s experience [32]. Thus, this design is particularly relevant to this study which aimed to explore the positive meaning of care work with older people and its implications for health care workers’ job satisfaction and motivation to work in the LTC sector.

In the context of Hong Kong, HCWs in LTC communities include personal care workers (PCWs) who take care of residents’ ADL and IADL, health workers (HWs, largely equivalent to “certified nursing assistants” in the United States) who monitor the work of PCWs and are responsible for the delivery of basic nursing care, and enrolled nurses (ENs) and registered nurses (RNs) who provide nursing care and oversee the work of PCWs and HWs.

### Recruitment sample

Purposive sampling was used to recruit HCWs from LTC communities as research participants. To meet the inclusion criteria, participants had to (1) be serving in the role of a PCW, HW, EN, or RN; (2) have at least 6 months of experience working in an LTC community; and (3) be providing frontline services to older people. The exclusion criteria were as follows: (1) LTC workers who had only a managerial role and did not provide frontline care; (2) LTC workers working in other roles (e.g., social workers, occupational therapists, physical therapists). In the process of participant recruitment, the maximum variation sampling method was used to ensure the heterogeneity of participants in terms of participants’ characteristics. The use of maximum variation sampling method aimed to recruit information-rich participants and to capture the widest range of possible perspectives [33]. Thus, in order to ensure maximum variation, this study recruited participants based on a variety of nature such as gender, age, role and rank, years of work experience, and types of LTC communities worked for including publicly subsidized and private communities.

Six LTC communities were approached by the researchers. The managerial staff of each LTC community was invited to refer potential participants to the researchers after briefed for the purpose of the study, as well as the inclusion and exclusion criteria of the sample. The researcher (S. Huang) liaised with the managerial staff to schedule the logistics. Participants were fully informed of the purpose and procedures of the study. Informed consents were obtained before data collection commenced. Pseudonyms were used in the study in order to protect participants’ identities.

Data were collected between February 2021 and December 2021. Thirty participants were recruited in the study. The average age of the participants was 37 years old, and their mean years of tenure in the care sector were 7 years. Reflecting the gender ratio of the overall population of the care workforce, 5 participants were male and 25 were female. Thirteen of the participants worked as nurses (five RNs and eight ENs), eight worked as HWs, and nine were PCWs. Sixteen participants had attained a post-secondary education and 13 had earned secondary education, with only one participant having received primary or below education (see Table 1 for demographic data of the participants) [see Additional file 1].

#### Data collection

Semi-structured in-depth interviews were conducted. Interviewers were trained in qualitative study methods and came from a variety of healthcare research backgrounds of nursing and social work. Interviews were conducted in private meeting rooms in LTC communities. Interview sessions lasted from 30 to 80 min (mean = 55 min). Cantonese was adopted in the interviews. An interview guide was developed for this study [see Additional file 2]. Each interview began with general questions revolving around the nature of the participant’s work and daily work routines, followed by exploratory questions that unraveled the meanings the participant made from her/his work. With the written informed consent of participants, all interviews were audio-recorded and transcribed verbatim.

### Data analysis

Thematic analysis [34] was used to analyze the interview data. Adopting an inductive approach to analysis, this study followed the six-phase approach to thematic analysis that includes (1) data familiarization, (2) coding, (3) initial theme generation, (4) theme development and review, (5) refining, defining and naming themes, and (6) writing up [35]. Two experienced qualitative researchers (V. Lai and S. Huang) coded each interview transcript independently. Transcripts were coded with the facilitation of the qualitative research data analysis software NVivo 12. All the authors met regularly to review interview transcripts, compare coding, and generate initial analytical themes together. Disagreements regarding coding were raised and discussed in team meetings until agreements were reached. Two authors then finalized the processes by developing, reviewing, refining, defining, and naming themes.

The trustworthiness and rigor of the study was ensured by credibility, dependability, confirmability and transferability [36]. In order to enhance the credibility, two researchers read the transcripts and conduct coding independently for comparison. They discussed the emergent themes and codes until a consensus was researched. Dependability was achieved by using an audit trail that detailed the description of the research process to reduce bias. Peer debriefing with an expertise was used for confirmability. Transferability of findings was attained by describing the participant characteristics and the methodology of the study transparently and comprehensively in order to allow readers understood the strengths and limitations of the study.

## Results

Engaging in care for others can be highly rewarding work as reflected from the participants. Five themes identified from the data that articulated the positive meaning that HCWs ascribed to their work in LTC communities, including (1) “My work makes their lives more comfortable”: Helping older people to age comfortably; (2) “Everyday our affections increase”: Building meaningful relationships; (3) “These are all skills”: Forming a professional identity of older people care; (4) “I want to find a job that ensures I will never be unemployed”: Ensuring job security; and (5) “They are extra work”: Barriers to attaining the positive meaning of work.

### “My work makes their lives more comfortable”: Helping older people to age comfortably

When making meaning of their work, the HCWs most frequently evoked the notion of helping older people to “age comfortably” in LTC communities. The idea of comfortable aging, as suggested by HCWs in this study, referred to both physical well-being (i.e., having desirable health outcomes and being free of pain) and psychosocial well-being. The physical and psychosocial well-being entailed the traditional socio-cultural values in Chinese society.

The HCWs suggested that their care activities supported older people’s comfortable aging by maintaining and even improving their physical health. The HCWs in LTC communities engaged in a variety of caregiving tasks in their everyday work. The daily work routine of the HWs, ENs, and RNs revolved around addressing the health needs of older residents through clinical and medical activities such as wound dressing, medication administration, peritoneal dialysis, tube feeding, etc. The care activities of the PCWs included personal care such as assisting with bathing, dressing, eating, toileting, transferring, grooming, etc., depending on the frailty level of the older residents. The HCWs suggested that they found their work meaningful because their care activities were helpful to older residents achieving desirable health outcomes.

I feel happy because my work makes their lives more comfortable. For example, a resident’s wound was quite severe and was at stage one or stage two before intervention. Then, we had multiple interventions and dressed the wound one shift after another until it finally healed. I gained a sense of fulfillment in the process. This process made me feel that our care was effective. (EN2)

As demonstrated by a participant, in the process of helping older people maintain their physical health, HCWs gain a strong sense of self efficacy and job satisfaction. Even though the HCWs pointed out that their care did not always lead to full recovery as many older people in LTC communities are physically frail and experiencing health deterioration, they deemed their work to be meaningful because it helped older people maintain the highest level of physical comfort possible.

Not everyone recovers. Some are not in a good condition, but at least my care helps to ensure they are not too bad. Even though they cannot recover fully, their wounds might get smaller or not deteriorate any more. They don’t feel so much pain… They can feel more comfortable. (HW3)

In addition, the HCWs suggested that their everyday care conveys companionship and psychological support to older people in LTC communities, which is also essential to their comfortable aging.

Actually, the meaning of taking care of them is about being part of their last journey of life. In other words, I can create a happy and comfortable later life for them before they pass away. There is someone who can talk with them and provide good care to them. For me, that is what nursing care is about. (EN4)

The idea of facilitating comfortable aging espoused by HCWs has socio-cultural relevance in Chinese society, where providing care to older people to enable their comfortable aging is seen as a moral virtue. Several HCWs, including those in younger ages, framed their care as rewarding and meaningful work as they believed that taking good care of older people would “accumulate good karma” for themselves and their family.

I quite like taking care of older people. It is like some sort of traditional thought… I think taking care of older people is accumulating good karma. I believe that this is beneficial to my family and myself. (EN1)

I think it is accumulating good karma. When taking care of older people, I am thinking that if I take good care of them now, I will be treated well by others when I get old and need care from others in the future. I do my work with this mindset. Therefore, I do not see my work as hard or dirty. (HW4)

### “Every day our affections increase”: Building meaningful relationships

The second theme that the HCWs ascribed to their work concerned the valuable long-term relationships they built with older residents in their daily work, through which they found joy and personal growth.

HCWs, especially the nurses, constantly drew comparisons between LTC communities and other health care settings, such as hospitals, when discussing the meaning of their work. They suggested that working in a LTC facility allowed them to form long-term, genuine bonds with the older people they cared for, something they argued was rarely possible elsewhere. According to a participant, residential homes allow “the cultivation of human relationships and affection that is absent in hospitals” (RN3). A participant further elaborated:

I like talking with people. Working in a hospital is like fighting a war. I had no time to know the backgrounds of my patients. I couldn’t even remember their names when they were discharged from the hospital. Then, I will never see them again… However, LTC communities are very different. The conditions of the older people we serve are more stable. I have more time to get along with them. (EN6)

The cultivation of relationship involves human interaction and emotional exchange as reflected from the participants. The HCWs believed that they were the ones who provided “close, personal care” to the residents. In the process of performing everyday care activities, they had frequent interactions and developed close relationships with older people. Many participants suggested that being able to communicate and interact with older people was the most enjoyable part of their work. Despite the challenges of caregiving work, participants found their relationships with older residents “joyful”, “satisfying”, and “rewarding”.

When I perform my work and provide care to them, I gain joy and fun out of it. I feel happy to interact with people. [The happiness] is very personal. It might be chatting with a resident and receiving an unexpected response. Some residents with dementia are very funny. They always come up with something unexpected and make me feel happy. (HW4)

The sense of satisfaction comes from my interactions with older people. Every day, our affections increase. They treat me like their granddaughter. I think acknowledgement from the boss does not matter a lot; I feel the biggest sense of satisfaction by getting the acknowledgement of the older people. They personally experience how well I deliver care. (EN5)

Moreover, some HCWs reported that their relationships with older residents were “reciprocal”, not only because they constantly received appreciation from the residents but also, more importantly, because they were able to learn “old wisdom” and achieve personal growth from the lived experiences of the older residents.

It is not only about providing a service to them; sometimes when I talk with them, they offer me their perspectives, from which I can learn something. This is more like a reciprocal relationship…Sometimes, the older people have old wisdom and special perspectives. (RN4)

I think I learn a lot from the older people because I meet a lot of people here and learn about their lived experiences from our conversations. They like sharing with me and I can reflect upon myself… (HW5)

### “These are all skills”: Forming a professional identity of older people care

HCWs proposed that older people care is highly skillful and professional, requiring communication, coordination, and chronic illness care skills. Being able to form a professional identity as a HCW for the older people thus constituted a salient MOW for the participants.

Participants in this study reported that they constantly experienced devaluation of their work by their family, friends, and health care professional allies, who regarded care work in LTC communities as “dirty, less skilled, and unprofessional”.

People imagine that this work is about changing diapers and dealing with shit and piss… My aunt used to say to me that she’d rather beg than work in a residential home. People are not willing to join this sector because they think older people care is dirty work and cannot accept dealing with human excreta. (PCW4)

They think that we work here because our nursing skills are not competent enough to work as hospital nurses. But when they hear that I am working in an LTC community, they doubt that my work is different from that in hospitals. They doubt that we work here because our nursing skills are not competent enough to work as hospital nurses. (EN6)

Contrary to the negative evaluations of their work, the HCWs evoked positive meanings of care work in LTC communities. One participant described that care in LTC communities and care in hospitals were “both part of the continuum of care that tackles the different health needs of older people, ranging from acute disease to long-term chronic illness” (RN5). More importantly, their care work in LTC communities allowed them to reimagine the nature of health care from delivering physical care tasks to providing holistic care that included psychological support, health education, human communication, resource coordination, and organizational management.

It is wrong to assume that nurses working with older people are not professional. Instead, we are differently professional in our specialties. For hospital nurses, their professional expertise lies in emergency treatment. But working in LTC is professional in terms of mastering the daily operation of a facility, governmental ordinances, and communication with family members. (RN2)

While the HCWs framed their work as valuable and professional, the HCWs described how performing personal care for older residents, such as positioning, lifting, transferring, feeding, and bathing, requires specialized knowledge, training, and experience.

Everything, every machine here requires specialized knowledge and training to handle. It is not that straightforward and simple. So, working as a PCW is not only about changing diapers. We need to grasp health and medical knowledge to monitor older peoples’ vital signs. We must also monitor whether the older people have bruises or wounds. We must be very careful to know whether the older people are doing ok. These are all skills. (PCW2)

Participants indicated that there were many other aspects that distinguished them as “professional” that further produced meanings and values in their personal life. One participant, a HW, indicated that working in LTC communities enabled her to work with interdisciplinary professionals such as doctors, nurses, nutritionists, social workers, physical therapists, and occupational therapists and thus allowed her to gain health knowledge. Many HCWs mentioned that the older people care knowledge and skills they learned from work could be useful in their personal life, particularly in terms of taking care of their older parents and grandparents at home.

### “I want to find a job that ensures I will never be unemployed”: Ensuring job security

HCWs, especially PCW and HW working in government-subsidized facilities, perceived that the LTC sector offers relatively promising job opportunities and security, a stable income, and a career development pathway. These instrumental values made the LTC sector attractive for the participants.

Across the globe, the LTC sector has long been suffering from the challenge of workforce shortage. For participants in this study, however, this challenge was perceived as a positive opportunity that added value to their jobs. Many proposed that they found older people care as meaningful work because with the trend of population aging, there would always be increasing workforce demands in the job market which could provide them with promising job opportunities and security. Some HCWs also mentioned that the job offered them income stability, which they deemed as valuable compared to other work in the service industry.

The availability of job stability and opportunities in older people care work was particularly salient for participants during the COVID-19 pandemic, when the unemployment rate was high due to economic recession. Several participants described that they joined the LTC sector during the COVID-19 pandemic for the stability it offered. For example, a participant described, “I was working in the hotel industry…Then I lost my job and couldn’t find a new one. I wanted to find a job that will ensure I will never be unemployed.” (PCW5).

In addition, participants suggested that they found their work meaningful because of the relatively promising career development opportunities. The LTC sector in Hong Kong provides HCWs with a career pathway and ladder to pursue career development. Although promotion and degree admission opportunities are highly competitive, some participants saw the career ladder that moves up from PCW, HW, and EN to RN as a promising pathway for them to gain better income and work benefits.

### “They are extra work”: Barriers to attaining meaning of work

Despite the HCWs ascribing a variety of positive meanings to their work, they admitted that it was not always possible to attain these meanings in their everyday work. They identified several barriers to attaining MOW, including the lack of organizational support for relational care, heavy workloads and workforce shortages, as well as emotional burnout.

As described above, HCWs found that the relational components of their work, particularly the helping relationships and affectional interactions with older residents, made the work highly meaningful. However, participants reported that although the LTC sector had long placed emphasis on person-centered care, they received little organizational support to develop meaningful relationships in their everyday work. Given that their daily work routines and timetables were predominantly organized around the delivery of physical caregiving tasks, many HCWs described an important and meaningful part of care work – relationship building and psychological support – as “extra” work that received little organizational recognition.

Of course, a lot of my work with the residents is extra work. I prefer to deliver holistic care that goes beyond physical care. Physical care tasks are those that appear on the timetable. But for the other parts, I must address them for the residents at other times by myself. (RN1)

Moreover, the heavy workloads and the chronic lack of workers in LTC communities impose further strains on HCWs in fulfilling their daily work routines, making it even more difficult for them to provide relational care. Despite these organizational constraints, the HCWs reported that they creatively made time and space in and between their work routines to build relationships and address older residents’ psychosocial well-being needs.

When I distribute medications, I usually have casual chats with the residents by greeting them and asking how their sleep and meals went. Just chatting. But it depends on the situation. When accidents happen, I would be too busy to handle this. (HW2)

Sometimes I am very busy and do not have time to interact with the residents at all… I usually use meal times when I am more or less available. Residents are usually sitting and waiting for meals before we distribute them. I will use the ten minutes or so to chat with them. (HW1)

Relationship building and affectional interaction can be satisfying and exhausting simultaneously. The HCWs described the high emotional demands from older people and their family members they had to bear in their everyday work, which frequently put them in a situation of emotional burnout which can detract from building meaning. In addition, some HCWs reported that it took a lot of emotional labor (i.e. to manage feeling as to fulfill job requirement) to care for older residents with difficult behaviors or personalities, especially those with declining mental health and dementia. They said that they constantly experienced distrust, blaming, and rejection from older residents when they performed caregiving tasks such as feeding, which added a considerable amount of strain to their work. Similarly, the HCWs had to deal with constant distrust and misunderstanding from residents’ family members, which caused some of them frustration and stress.

This is work that cannot get understanding from everyone. Some [family members of the residents] would not notice my efforts to care for the residents. However, if I make a minor mistake, they will blame me. Human beings make mistakes and are not perfect. I am also sincerely concerned for the older people, but they don’t understand and blame me for my mistake. (PCW7)

## Discussion

This study examines HCWs’ engagement in meaningful work in LTC communities in the context of an economically developed Chinese society in Hong Kong. It is found that HCWs deemed their work to be a meaningful helping career that facilitated comfortable aging for older people and connoted positive socio-cultural values. They further attributed their MOW to the valuable relationships developed in their daily work and to the positive professional identity and relatively promising job security in their work, although the attainment of positive MOW was hindered by a number of barriers. In this discussion, we describe how these findings can support social policy initiatives to attract, retain, and support the LTC workforce.

To date, research and social policy interventions on LTC workforce development have largely focused on structural factors that influence the retention of HCWs and their job satisfaction [37]. Studies informed by this line of inquiry have identified the importance of working conditions, especially pay and compensation, workload and staffing level, teamwork, and supervision, in shaping work-related outcomes [29, 38, 39]. Even though the positive organizational scholarship has long argued the beneficial impacts of positive psychological states, including perceptions of meaningful work, on workforce functioning and productivity [40], relatively little attention has been paid to positive working experiences in the LTC sector. Our study moves a step forward from the current literature by shedding light on the subjective meaning making of work as an important, yet often overlooked, aspect of direct care work in LTC communities. While the structural factors of working conditions are pivotal to the job quality in LTC communities, MOW can serve as a psychological resource that engenders positive emotions and motivates HCWs to engage in direct care work in LTC communities. The findings of this study thus provide nuanced evidence about promoting meaningful work as a promising intervention for LTC workforce development. This could be done by addressing structural factors such as promoting job security, improving time and resource constrains, enhancing organizational support in LTC communities. Also, this could be done by supporting relationship building and better integrating psychosocial care into older people care work and exploring socio-cultural resources that contribute to positive meaning making of older people care work. In addition, as an extension of this qualitative study, quantitative research that examines the impacts of MOW on workers’ turnover intention and job satisfaction, as well as MOW as a mediating mechanism in explaining the impacts of working conditions on worker outcomes in the LTC sector, will be an important area for future exploration.

The findings of this study also imply that the meaning construction of older people care should be further understood and supported in the broader contexts, including the LTC policy, organizational support, and the socio-cultural meaning of older people care. As indicated by our research findings, the professional identity and job security in the LTC sector are important parts of HCWs’ MOW. While research to date has stressed the lack of job security and professional status in the LTC sector [41], our study has provided somewhat contradictory findings. Participants in the present study has relatively positive perceptions about career prospects in the LTC sector, proposing that the growing demand for LTC in the face of population aging entails job opportunities and job security, both of which make a career in LTC attractive. The nurses highlighted that their work was different to but equally as professional, skilled, and challenging as acute hospital care. Some indicated that their nursing care experiences in LTC communities allowed them to develop specialties in chronic disease management to maintain the wellness and quality of life of older people. This positive perception of LTC work is partly shaped by the preliminary, yet far from finished, social policy attempts to professionalize the LTC workforce in the local context. In Hong Kong, LTC policy has laid out the foundation of a relatively promising career development pathway in the nursing profession for HCWs in the LTC sector, most noticeably through the establishment of the Vocational Qualifications Pathway (VQP) for the LTC service industry and professional training programs [42]. Our findings thus call for research and social policy interventions to address the professionalization of the LTC sector and enable HCWs to gain public recognition, rewarding pay, job security, and career development.

Additionally, the findings of this study add to the existing studies on working conditions in LTC communities by highlighting the lack of organizational support for relational care as an organizational barrier to attaining meaningful work. Our study echoes existing research findings that HCWs deem affectional interactions and long-term relationships with older people as meaningful and valuable [29]. Yet HCWs’ yearning for meaningful relationships with older people is constantly constrained by the organizational structures of LTC communities, particularly the traditional institutional model of care centered around measurable and functional caregiving tasks [43, 44]. The culture change movement that calls for humanizing care practice by transforming the institutional form of care in LTC communities to person-centered and relational care [45, 46]. This culture change movement is thus particularly relevant to promoting the meaningful work of HCWs. Facilitating positive, meaningful working experiences for the LTC workforce would require changes in the organizational cultures of LTC communities to enable flexible caregiving routines, professional training opportunities that address relationship and rapport building, and a humanizing working environment.

Lastly, the meaning of older people care is constructed under an array of socio-cultural values. Even though increasing scholarly attention is being paid to revealing a culturally sensitive approach to older people care [47], very few studies have examined the socio-cultural meanings and values attached to older people care work from HCWs’ perspectives in the international contexts. As illustrated in this study, the notion of facilitating comfortable aging was seen as “accumulating good karma” and contained socio-cultural meaning towards older people care within the Chinese society. While engaging in older people care work is socially constructed as a “dirty work” [17], it could entail cultural salience and be regarded as a rewarding career in a society that values the life experience and moral authority of older people. This finding thus reveals the importance of a culturally sensitive perspective in researching and developing social policy interventions for LTC workforce development, including promoting a culturally resonant positive image of work in the LTC sector. This policy implication is not only resonant to other Asian societies, but also to the international contexts as Asian migrant workers represent a considerable proportion of the LTC workforce in developed countries such as Australia, US, UK and other European countries [48, 49]. 

### Limitations

Although this study adopted the maximum variation sampling method to increase the variety of HCWs’ perspectives and experiences, its use of purposive sampling is limited in representativeness. Additionally, this research intended to explore the MOW for all types of HCWs (eg, EN, RN, HW, PCW). However, these HCWs have quite different working experiences and work meaning because of different job quality and professional status. As non-nurses are particularly vulnerable to the deprivation of subjective well-being in work because of the poor job quality of their work [5], future studies would benefit from examining the subjective meaning making of work among this specific group of workers.

## Conclusion

This qualitative study explores how HCWs negotiate the positive meaning of older people care work and the implications of meaningful work for workers’ job satisfaction and motivation to work in the LTC sector in Hong Kong’s LTC communities. While HCWs perform physically and emotionally demanding care work, they actively construct a subjective meaning of older people care as a helping career that enables them to facilitate comfortable aging of older people, build affectionate relationships, achieve professional identity, and gain job security. Their construction of meaningful work is further discussed in an array of social policy, organizational, and socio-cultural factors that all entail future research and social policy implications of LTC workforce development.

### Supplementary Information

Supplementary Material 1.

Supplementary Material 2.

## Data Availability

The datasets generated and analysed during this study are not publicly available to protect the participant' confidentiality. However, they are available from the corresponding author upon reasonable request.

## Abbreviations

ADL: Activities of daily living
ENs: Enrolled nurses
HCWs: Health care workers
HWs: Health workers
IADL: Instrumental activities of daily living
LTC: Long-term care
MOW: Meaning of works
PCWs: Personal care workers
RNs: Registered nurses
