# Supplementary material for: Health care workers’ self-perceived meaning of residential care work
Source: BMC Health Serv Res. 2024 Jun 26;24:766. doi: 10.1186/s12913-024-11218-2 (PMC11201782; doi:10.1186/s12913-024-11218-2)
Supplement: Supplementary file 1 — Supplementary Material 1. [file 12913_2024_11218_MOESM1_ESM.docx]

**Table 1.** Demographic data of the participants

| **Case number** | **Role*** | **Age** | **Gender** | **Education** | **Years of working in the residential care sector** |
| --- | --- | --- | --- | --- | --- |
| 1 | EN | 28 | M | Post-secondary | 2 |
| 2 | HW | 53 | F | Post-secondary | 11 |
| 3 | EN | 31 | F | Post-secondary | 5.5 |
| 4 | RN | 36 | M | Post-secondary | 10 |
| 5 | PCW | 45 | F | Primary and below | 5 |
| 6 | PCW | 52 | F | Secondary | 11.5 |
| 7 | EN | 49 | F | Secondary | 18 |
| 8 | PCW | 33 | F | Secondary | 3 |
| 9 | EN | 27 | F | Post-secondary | 1.5 |
| 10 | EN | 32 | F | Post-secondary | 1.5 |
| 11 | PCW | 21 | F | Secondary | 0.5 |
| 12 | PCW | 59 | F | Secondary | 20 |
| 13 | PCW | 31 | F | Post-secondary | 10 |
| 14 | RN | 34 | F | Post-secondary | 5.5 |
| 15 | HW | 18 | M | Secondary | 1 |
| 16 | EN | 27 | F | Post-secondary | 2.5 |
| 17 | EN | 35 | F | Post-secondary | 7 |
| 18 | RN | 56 | F | Post-secondary | 12 |
| 19 | PCW | 30 | F | Secondary | 0.5 |
| 20 | HW | 30 | M | Secondary | 1 |
| 21 | EN | 24 | F | Post-secondary | 1.5 |
| 22 | HW | 21 | F | Post-secondary | 2 |
| 23 | PCW | 58 | F | Secondary | 1.5 |
| 24 | HW | 43 | F | Post-secondary | 16 |
| 25 | PCW | 21 | F | Secondary | 3 |
| 26 | HW | 49 | F | Secondary | 20 |
| 27 | RN | 28 | F | Post-secondary | 5 |
| 28 | HW | 69 | F | Secondary | 25 |
| 29 | HW | 55 | F | Secondary | 6 |
| 30 | RN | 28 | F | Post-secondary | 1 |

* Role: RN=Registered Nurse; EN=Enrolled Nurse; HW=Health Worker; PCW=Personal Care Worker.
