# Supplementary material for: Health care workers’ self-perceived meaning of residential care work
Source: BMC Health Serv Res. 2024 Jun 26;24:766. doi: 10.1186/s12913-024-11218-2 (PMC11201782; doi:10.1186/s12913-024-11218-2)
Supplement: Supplementary file 2 — Supplementary Material 2. [file 12913_2024_11218_MOESM2_ESM.docx]

**Interview Guide**

1. Please briefly introduce the work you are responsible for in the long-term care facilities / residential care home for the elderly.
2. What do you like about your job?
3. What do you like least about your job?
4. What does the meaning of work of caring for the older people mean to you? What motivates you to continue doing this job?
5. How do you think your family, friends and society view the work you do?
6. In summary, do you consider your job to be “dirty work”? Why?
